# The effect of cognitive behavioral therapy for sleep and glycemic outcomes in people with diabetes: A systematic review and meta-analysis

**DOI:** 10.1097/MD.0000000000050890

**Published:** 2026-10-09

**Authors:** Yuxiao Jiao, Xiaofei He, Lulu Ma, Bo Zhang, Yueqi Yin, Lanyu Zhu

**Affiliations:** a School of Nursing, Changchun University of Chinese Medicine, Changchun, Jilin Province, China; b Nursing Department, First Teaching Hospital of Tianjin University of Traditional Chinese Medicine, Tianjin, China.

**Keywords:** cognitive behavioral therapy, diabetes, glycemic outcomes, Meta-analysis, sleep

## Abstract

**Background:**

Sleep disturbances are highly prevalent in individuals with diabetes and are associated with various adverse physiological, psychological, and social consequences. A growing body of research supports the effectiveness of cognitive behavioral therapy (CBT) in managing sleep problems in diabetes. However, the extent of its positive correlation with sleep quality and glycemic outcomes remains unclear due to the lack of rigorous evidence synthesis.

**Methods:**

Ten databases (PubMed, Cochrane Library, EMBASE, CINAHL, Web of Science, MEDLINE, APA PsycInfo, China National Knowledge Infrastructure [CNKI], Wanfang Data Knowledge Service Platform, VIP) were searched using keywords related to cognitive behavioral therapy and diabetes, from their inception until June 2026, to identify eligible randomized controlled trials. Researchers independently screened and extracted data from the included studies. The risk of bias was assessed using the Cochrane Risk of Bias tool (RoB 2.0). Random-effects meta-analyses were performed using Review Manager software (RevMan) 5.4.1 to estimate overall effects and for prespecified subgroup analyses. Sensitivity analysis, assessment of publication bias, and meta-regression were conducted using STATA 17.0 to explore study-level variables potentially contributing to heterogeneity.

**Results:**

Ten eligible randomized controlled trials involving a total of 2827 participants from 4 countries were included in this synthesis. When comparing CBT to usual care or health education, CBT demonstrated a significant effect size for improving sleep quality (mean difference [MD] = −1.47; 95% confidence interval [CI] = −1.98 to −0.96; *P* < .00001; *I*^2^ = 89%). Significant improvements were also observed for glycemic outcomes (HbA1c: MD = −0.35%; 95% CI = −0.44 to −0.25; *P* < .00001; *I*^2^ = 94%) and insomnia severity (MD = -2.06; 95% CI = −4.24 to 0.12; *P* = .004; *I*^2^ = 88%). However, there was no significant improvement in BMI (MD = −0.12; 95% CI = −0.72 to 0.48; *P* = .69; *I*^2^ = 0%). Exploratory subgroup analyses revealed significant differences based on follow-up time. Through meta-regression, disease type, sample size, and intervention plan were not found to be sources of heterogeneity.

**Conclusion:**

Cognitive behavioral therapy significantly improves sleep quality, reduces HbA1c levels, and alleviates insomnia severity in people with diabetes. Future high-quality research is needed to optimize protocols, define target populations, and facilitate wider clinical implementation.

## 1. Introduction

Diabetes mellitus (DM) is a chronic metabolic disorder characterized by hyperglycemia resulting from defects in insulin secretion, insulin action, or both. It is primarily classified into type 1, type 2, and gestational diabetes.^[1]^ By 2022, the global prevalence of diabetes among adults aged ≥ 18 years surged from approximately 200 million in 1990 to 828 million, with the most significant increase occurring in low- and middle-income countries.^[2,3]^ Sleep disturbances in individuals with diabetes represent complex symptomology, encompassing impaired sleep quality and/or abnormal sleep duration. People with diabetes are particularly susceptible to developing sleep disturbances and experiencing severely diminished sleep quality due to various complications and the impact of negative psychological states like anxiety and depression. Research indicates that the prevalence of comorbid sleep disturbances in diabetes is approximately 47.2%, consistent with findings by Kodakandla et al.^[4,5]^

Sleep disturbances can inhibit insulin secretion and increase the secretion of counter-regulatory hormones, exacerbating insulin resistance and consequently elevating blood glucose levels.^[6,7]^ Sleep is also modulated by glucocorticoids, implicating the hypothalamic-pituitary-adrenal (HPA) axis in the relationship between sleep quality and cognitive function; prolonged poor sleep quality can lead to reduced cognitive function.^[8,9]^ Furthermore, it may also contribute to the development of mental disorders and cardiovascular/cerebrovascular diseases.^[10,11]^

Current management of sleep problems in diabetes often involves pharmacotherapy, including hypoglycemic agents and sedative-hypnotic drugs.^[12]^ However, pharmacological interventions are associated with numerous adverse effects, such as circulatory and respiratory depression, drowsiness, gastrointestinal motility dysfunction, and the potential for tolerance and dependence with long-term use. They may also interact adversely with anesthetic agents used during surgery.^[13]^ These limitations have prompted healthcare professionals to investigate complementary and alternative medicine approaches, defined as healthcare materials and practices not classified as conventional medicine.

Cognitive Behavioral Therapy, as a psychological intervention, is a psychotherapeutic approach that aims to correct cognitive distortions through cognitive restructuring, thereby eliminating negative emotions and promoting behavioral adjustments.^[14]^ First proposed by Dr Aaron Beck in the US in the 1960s, it has been widely applied in the treatment of various psychological disorders with demonstrated efficacy. Numerous national and international guidelines endorse cognitive behavioral therapy for insomnia (CBT-I) as the first-line treatment for insomnia disorder.^[15,16]^ CBT-I targets modifying patients’ belief systems and irrational beliefs/attitudes about insomnia, enhancing their confidence in self-managing sleep difficulties. Core components include sleep hygiene education, stimulus control therapy, sleep restriction therapy, relaxation training, and cognitive therapy. Relevant studies suggest that compared to pharmacotherapy alone, CBT-I is less costly and offers greater cost-effectiveness, particularly in patients with comorbid psychiatric conditions and the elderly. It also reduces adverse drug effects by decreasing medication use.^[17]^ The efficacy of CBT has been substantiated by extensive clinical research, showing benefits for chronic insomnia in the elderly, post-stroke insomnia, and insomnia comorbid with depression.

The core principle of CBT lies in correcting erroneous cognitions and beliefs about sleep held by individuals with insomnia. Patients often experience negative automatic thoughts involving excessive worry about sleep loss and its consequences, which perpetuate sleep difficulties;^[18]^ individuals with diabetes frequently experience anxiety and depression due to disease-related stress and poor glycemic control, which further exacerbate insomnia. CBT helps patients identify and modify these negative thought patterns, improving emotional states, thereby alleviating anxiety and depression and subsequently enhancing sleep quality.^[19,20]^ Furthermore, CBT addresses maladaptive sleep habits and behaviors, educates patients on scientific sleep principles, and helps them understand the relationship between insomnia and diabetes, thereby improving self-management capabilities and sleep quality.^[21]^ Importantly, CBT not only improves sleep but also exerts a positive influence on glycemic control. By reducing chronic stress responses and lowering inflammatory markers, it indirectly improves metabolic function and glucose regulation.^[21]^

While existing research suggests the effectiveness of CBT for sleep in diabetes, and 1 meta-analysis found CBT beneficial for improving sleep in this population.^[21,22]^ these analyses focused solely on sleep-related outcomes and did not examine changes in glycemic indices or other metrics. Furthermore, certain databases were not included in their search strategies, potentially excluding relevant evidence. Considering the limitations of previous meta-analyses and the inconsistent findings regarding CBT’s efficacy in prior trials, this systematic review and meta-analysis aims to synthesize and evaluate the evidence concerning the effects of CBT on both sleep quality and glycemic outcomes in people with diabetes.

## 2. Materials and methods

### 2.1. Protocol registration

The protocol for this review was registered on PROSPERO (Registration number: CRD420250654106).

### 2.2. Search strategy

Ten databases (PubMed, Cochrane Library, EMBASE, CINAHL, Web of Science, MEDLINE, APA PsycInfo, CNKI, Wanfang, VIP) were searched for relevant journal articles published from their inception until June 2026. Search strategies utilized combinations of keywords and MeSH terms such as: “Cognitive Behavioral Therapy”[Mesh] OR “Behavioral Therapies, Cognitive”[Title/Abstract] OR “Behavioral Therapy, Cognitive”[Title/Abstract] AND “Diabetes Mellitus”[Mesh] OR “diabetes mellitus*”[Title/Abstract] OR “diabetes”[Title/Abstract] OR “glycuresis”[Title/Abstract] AND “randomized controlled tria”l[Publication Type] OR “randomized”[Title/Abstract]. After 2 reviewers (JYX, HXF) independently screened titles and abstracts, full-text assessment based on eligibility criteria was performed independently by the same 2 reviewers. Any disagreements were resolved by a third reviewer (MLL).

### 2.3. Inclusion criteria

Article inclusion criteria were defined using the PICOS framework: Subjects: Target subjects were adults (age ≥ 18 years) diagnosed with diabetes. Intervention: The intervention group received CBT, either alone or alongside usual care. Comparator: Comparator groups included usual care, waitlist control, placebo, or health education. Study outcome: Primary outcome: Sleep quality, measured using the Pittsburgh Sleep Quality Index (PSQI). Secondary outcomes: Glycemic control, body mass index (BMI), insomnia severity. Study design: Randomized controlled trials (RCTs).

### 2.4. Exclusion criteria

Full text not available in English or Chinese and studies involving subjects with primary psychiatric disorders.

### 2.5. Data extraction

Data were extracted by 2 reviewers using a predefined form, including: author, publication year, population characteristics, sample size, participant mean age, follow-up duration, study aim, details of interventions in CBT and control groups, intervention duration, intervention frequency, and outcome measures. The primary outcome for this systematic review was sleep quality assessed at the first evaluation post-intervention. Secondary outcomes included HbA1c, BMI, and insomnia severity post-intervention.

### 2.6. Risk of bias assessment

The methodological quality of included articles was assessed using the Cochrane Risk of Bias tool (RoB 2.0). Five domains were evaluated: the randomization process, deviations from intended interventions, missing outcome data, measurement of the outcome, and selection of the reported result. Each domain was rated as “low risk,” “some concerns,” or “high risk.” An article was classified as: “low risk” if all domains were rated “low”; “Some concerns” if at least 1 domain was rated as “some concerns”; “High risk” if at least 1 domain was rated “high risk.”^[23]^ The certainty of evidence was also classified using the GRADE framework as high, moderate, low, or very low.^[24]^ Disagreements during assessment were resolved by a third reviewer. Trials were included regardless of risk of bias.

### 2.7. Statistical analyses

Data analysis was performed using Review Manager software (RevMan) 5.4.1 (The Cochrane Collaboration) and Stata 17 (StataCorp LLC). When outcomes were measured using the same scale across trials, the overall effect size was calculated as standardized mean difference (SMD) with 95% confidence intervals (CI). *P* < .05 was considered statistically significant. Heterogeneity was assessed using Cochran *Q* test, with the *I*^2^ statistic representing the proportion of between-study variability. *I*^2^ values of 25%, 50%, and 75% indicated low, moderate, and high heterogeneity, respectively.^[24]^ A random-effects model was used if I^2^ > 50%; otherwise, a fixed-effects model was applied.^[25]^ Subgroup analyses were performed for sleep quality and HbA1c outcomes to investigate sources of heterogeneity and the efficacy of CBT based on different follow-up times and intervention durations.

If publication bias existed, the trim-and-fill method was performed to adjust for it, which involves trimming studies that caused asymmetry in the funnel plot and filling imputed missing studies in the funnel plot to address the publication bias. Univariate meta-regression was conducted to investigate the association between sleep quality and participant characteristics (type of disease), sample size, and intervention.

## 3. Results

### 3.1. Literature search results

The stages of electronic search, identification, and screening of eligible studies are described in the PRISMA flowchart. The initial search strategy yielded 2319 studies. After removing duplicates and screening titles/abstracts, studies were retained for full-text assessment. Ultimately, 10 studies involving 2827 subjects were included in this systematic review and meta-analysis.^[26-35]^ The screening process and results are shown in Figure 1.

**Figure 1.**
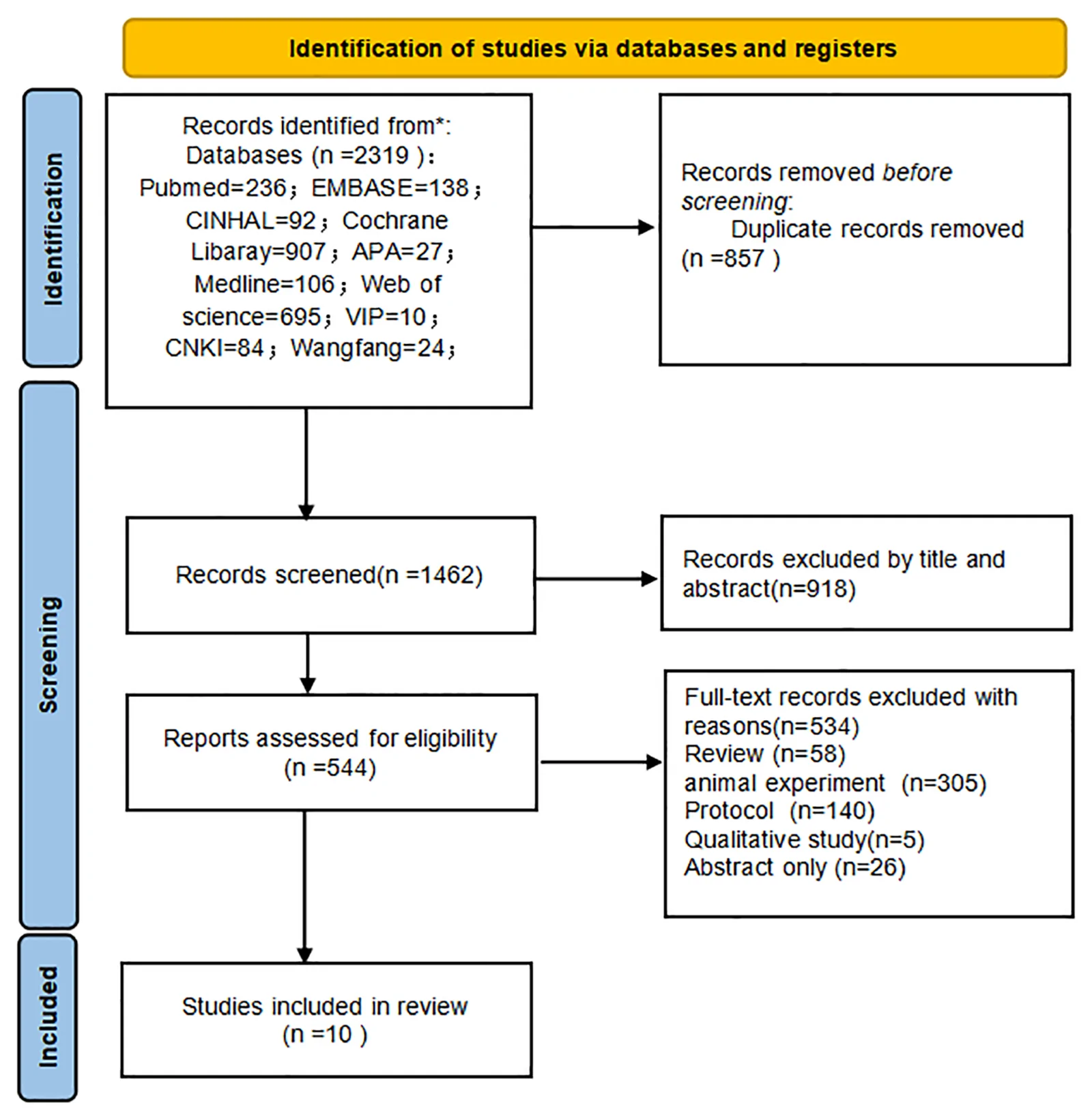
Flow diagram summarizing the selection process of meta-analyses.

### 3.2. Characteristics of included studies

The studies originated from China (n = 7), the United States (n = 1), Iran (n = 1), and the Netherlands (Amsterdam, n = 1). Sample sizes ranged from 28 to 1142. Participants in all included studies were adults (≥18 years), with mean ages ranging from 29.17 to 71.8 years. Five studies included general diabetes populations, 2 included diabetes patients with comorbid insomnia, 1 included gestational diabetes patients, 1 included diabetic patients with postpartum depression, and 1 included diabetes patients with comorbidities. Sleep quality was assessed using validated scales: PSQI (n = 9). Glycemic control was assessed using HbA1c (n = 8). Detailed characteristics of the included studies are presented in Tables 1 and 2.

**Table 1:** General description of the characteristics of each meta-analyses.

Author (yr)	Intervention	Control	Duration	Frequency	Outcome	Main outcome	Intergroup differences
Zuo et al (2020)^[26]^	CBT with aerobic exercise plus UC	Usual care (UC)	2 mo	One lecture plus a discussion session each week for 7 wk	①②③	Sleep Quality	*P* < .001
Lenka et al (2024)^[27]^	Online CBT-I e-health program	Usual care	5-wk	A 5-week online CBT-I e-health program, Face-to-face education	①②④⑤⑥⑦⑧	Sleep Quality; HbA1c	*P* < .01; *P* > .05
Mohammed (2020)	CBT-I	Health education	6 wk	One session per week, six sessions of CBT-I	①②③④⑨⑩	Sleep Quality; HbA1c	*P* = .01; *P* = .02
Zhang (2021)	CBT + usual care	Usual care	7 wk	Everyday in the first week, 2 lectures plus discussion sessions each week for 6 wk	①②	Sleep Quality; HbA1c	*P* < .001; *P* < .001
Dong (2020)	CBT	Face-to-face education	8 wk	Once a week, 8 sessions	①②	Sleep Quality; HbA1c	*P* < .01; *P* < .01
Li (2023)	CBT	Usual care	6 mo	First 2 mo: 8 weekly sessions (1 session per week). Following 4 mo: 4 monthly sessions (1 session per month).	①②⑧	Sleep Quality; HbA1c	*P* = .027 *P* = .863
Chen (2024)	CBT group	Hypoglycemic group	12 wk	Once a week, the intervention lasts for 12 wk	①②	Sleep Quality; HbA1c	*P* = .0401; *P* < .01
Wu (2023)	Sertraline + behavioral cognitive therapy	Sertraline	4 wk	NA	①	Sleep Quality	*P* < .05
Li (2018)	Diabetes sleep education, combined with conventional diabetes education	Diabetes education	6 wk	6 sessions	①②⑧	Sleep Quality; HbA1c	*P* < .001; *P* < .05

① Pittsburgh Sleep Quality Index (PSQI), ② HbA1c, ③ Diabetes-Specific Quality of Life Scale (DSQLS), ④ Insomnia Severity Index (ISI), ⑤ Sleep Onset Latency (SOL), ⑥ Sleep Efficiency (SE), ⑦ Wake After Sleep Onset (WASO), ⑧ Body mass index (BMI), ⑨ Epworth daytime sleepiness scale (ESS), ⑩ Sleep Self-Efficacy Scale (SSES).

CBT = cognitive behavioral therapy, CBT-I = cognitive behavioral therapy for insomnia, UC = usual care.

**Table 2:** Primary studies included in meta-analyses.

Author (yr)	Country	Participants	Number of participants	Mean age ± SD	Follow-up time	Aim
Zuo (2020)	China	Type 2 diabetes mellitus (T2DM)	Total:187 IG:94 CG:93	IG:63.9 ± 10.2 CG:61.7 ± 10.4	6 mo	To test the effect of CBT on sleep disturbances and QoL in patients with T2DM
Lenka (2024)	Amsterdam	People with type 2 diabetes and insomnia symptoms	Total: 57 IG:29 CG:28	IG:67.7 ± 8.4 CG:71.8 ± 7.3	3 mo 6 mo	Investigate whether aiding sleep by online cognitive behavioral therapy for insomnia (CBT-I) can improve glycemic and metabolic control, mood, quality of life (QoL) and insomnia symptoms in people with type 2 diabetes
Mohammed (2020)	US	People with T2D and insomnia symptoms.	Total:28 IG:14 CG:14	IG:61.86 6.48 CG:59.43 9.49	NA	To examine the effect of cognitive behavioral therapy for insomnia (CBT-I) on the severity of insomnia in people with type 2 diabetes
Zhang (2021)	China	Type 2 diabetes mellitus	Total:1142 IG:568 CG:574	IG:61.58 ± 9.17 CG:61.83 ± 8.64	2 mo 6 mo 12 mo	To test the effect of CBT delivered by general practitioners in improving sleep quality and reducing glycemic levels in patients with T2DM in community
Dong (2020)	China	Type 2 diabetes mellitus (T2DM)	Total:852 IG:412 CG:440	IG:64.35 ± 9.89 CG:63.06 ± 9.70	6 mo 12 mo	To explore the effect of cognitive behavioral therapy on sleep and HbA1c in type 2 diabetes patients
Li (2023)	China	Type 2 diabetes (T2DM) patients with comorbidities	Total:281 IG:148 CG:133	IG:47.66 ± 12.31 CG:52.4 ± 10.80	NA	to investigate the effectiveness of CBT on psychological variables, behavior variables, quality of life, sleep quality, and physical variables among adult T2DM patients with comorbid metabolic syndrome (MS)
Chen (2024)	China	Gestational diabetes mellitus	Total:96 IG:48 CG:48	IG:29.77 ± 3.96 CG:29.81 ± 3.63	NA	To explore the application of hypoglycemic drugs combined with cognitive behavioural therapy in patients with gestational diabetes
Wu (2023)	China	Pregnant women with diabetes complicated with postpartum depression.	Total:90 IG:45 CG:45	IG:29.17 ± 3.69 CG:29.52 ± 3.48	NA	To explore the clinical effect of behavioral cognitive therapy combined with sertraline on pregnant women with diabetes complicated with postpartum depression
Li (2018)	China	Type 2 diabetic patients	Total:31 IG:15 CG:16	IG:53.7 ± 17.2 CG:51.6 ± 12.2	3 mo	To investigate whether individualized diabetes sleep education significantly improve sleep quality and glycemic control in type 2 diabetic patients

CBT = cognitive behavioral therapy, CBT-I = cognitive behavioral therapy for insomnia, MS = metabolic syndrome, QoL = quality of life, T2DM = Type 2 diabetes mellitus.

### 3.3. Quality appraisal of included studies

A summary of the risk of bias assessment is shown in Figure 2. Most studies described the random sequence generation process (n = 8, 80%), but only 2 described allocation concealment measures (20%). Blinding of participants was difficult or impossible due to the nature of the CBT intervention and follow-up, resulting in “some concerns” or “high risk” ratings for deviations from intended interventions in most studies. Only 2 studies reported blinding of outcome assessors. A low risk of bias due to missing outcome data and in selection of the reported result was observed in all 10 studies. No other sources of bias were identified. The quality evaluation of the included studies is shown in Figures 2 and 3.

**Figure 2.**
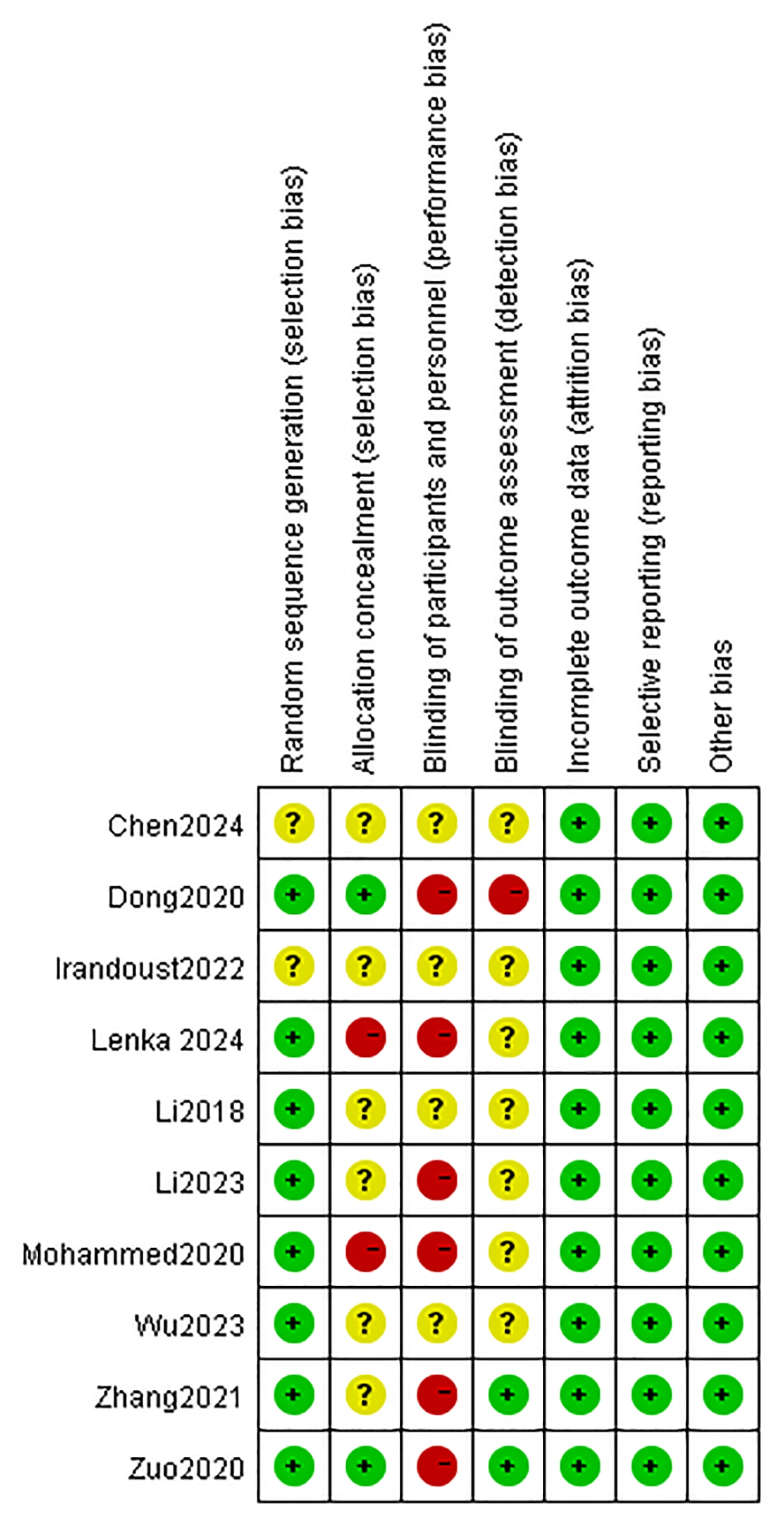
Risk of bias summary graph.

**Figure 3.**
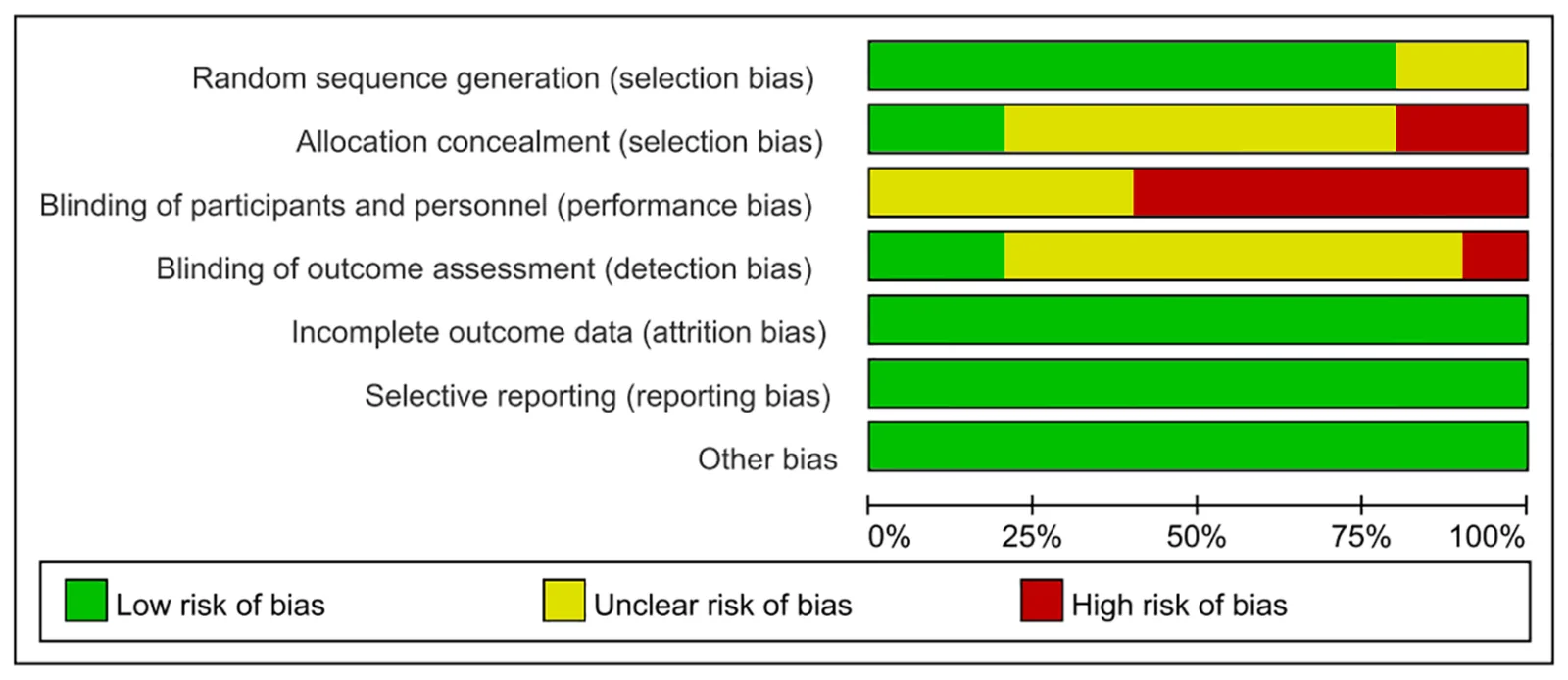
Risk of bias graph.

### 3.4. Results of the meta-analysis

#### 3.4.1. Effectiveness of CBT on sleep quality at different follow-up time points

Nine studies assessed the effect of CBT intervention on patient sleep quality compared to control groups.^[26-34]^ All studies reported statistically significant improvements in sleep quality (*P* < .05). The pooled results indicated that CBT intervention had a statistically significant effect on improving sleep quality compared to usual care or health education (SMD = −1.28; 95% CI = −1.74 to −0.82; *P* < .00001; *I*^2^ = 91%).

Subgroup analysis was performed according to follow-up time. Four studies assessed sleep quality immediately post-intervention. CBT showed a statistically significant improvement compared to control (SMD = −1.43; 95% CI = −2.05 to −0.81;*P* = .006).^[28,31-33]^ Heterogeneity changed upon removal of Chen et al (2024), possibly related to its specific population (gestational diabetes). At the 3-month follow-up (n = 2 studies),^[27,34]^ no statistically significant difference was found between CBT and control groups (*I*^2^ = 64%); sensitivity analysis was not performed due to the small number of studies. At the 6-month follow-up (n = 4 studies), *P* = .10,^[26,27,29,30]^ CBT showed a statistically significant large improvement in sleep quality compared to control (SMD = −1.42; 95% CI = −2.71 to −0.13; *P* < .00001; *I*^2^ = 97%); heterogeneity remained high upon sequential removal of studies. Similarly, at the 12-month follow-up (n = 2 studies),^[29,30]^ CBT showed a statistically significant large improvement (SMD = −1.39; 95% CI = −1.74 to −0.82; *I*^2^ = 92%); sensitivity analysis was not performed due to the small number of studies (Fig. 4).

**Figure 4.**
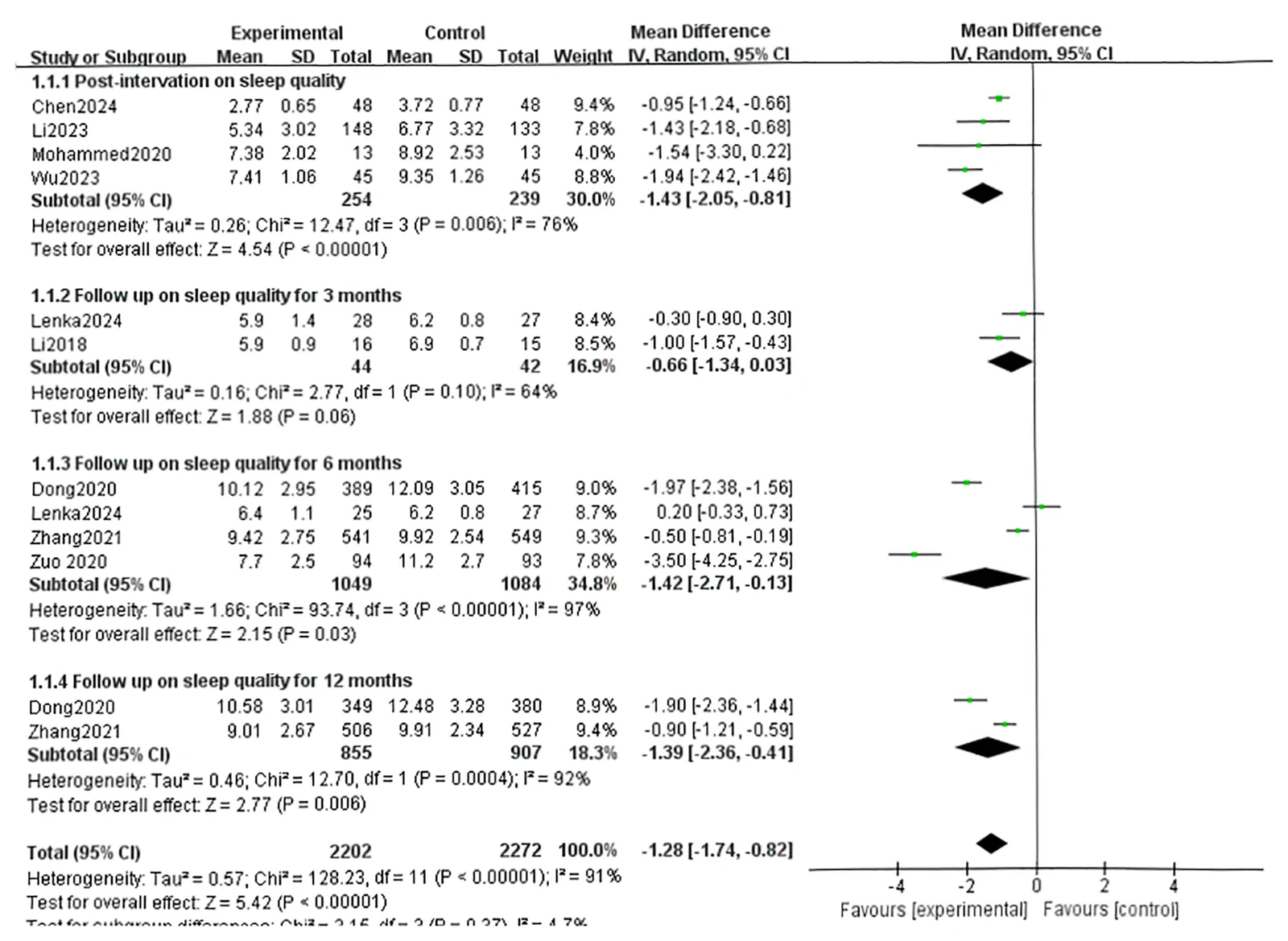
Forest plot of CBT effect on sleep quality (PSQI) by follow-up time. CBT = cognitive behavioral therapy, CI = confidence intervals, PSQI = Pittsburgh Sleep Quality Index, SD = standard deviation.

#### 3.4.2. Effectiveness of CBT on HbA1c at different follow-up time points

Eight studies assessed the effect of CBT intervention on HbA1c levels.^[26-32,34]^ Except for 1 study reporting no statistically significant difference in HbA1c (*P* = .863),^[31]^ all other studies reported statistically significant reductions compared to control (*P* < .05). The pooled results indicated a statistically significant moderate effect of CBT intervention on reducing HbA1c compared to usual care or health education (SMD = -0.30; 95% CI = −0.37 to −0.24;*P* < .00001; *I*^2^ = 90%). Heterogeneity remained high when each study was removed sequentially.

The subgroup analysis followed the same sleep quality assessment protocol as the main study. Three studies conducted HbA1c measurements immediately after intervention, revealing statistically significant improvements in the CBT group compared to the control group (SMD = −0.34; 95% CI = −0.46 to −0.22; *P* < .00001; *I*^2^ = 98%).^[28,29,32]^ At 3-month follow-up (n = 3), no statistically significant differences were observed between groups (*P* = .59, *I*^2^ = 0%).^[27,31,34]^ However, a marked improvement in HbA1c levels was detected at 6-month follow-up (SMD = −0.30; 95% CI = −0.41 to −0.19; *P* = .004, *I*^2^ = 78%; n = 4).^[26,27,29,30]^ Subgroup analysis at 12-month follow-up demonstrated further progression (SMD = −0.30; 95% CI = −0.37 to −0.24; *P* < .00001; *I*^2^ = 37%; n = 2).^[29,30]^ The results are shown in Figure 5.

**Figure 5.**
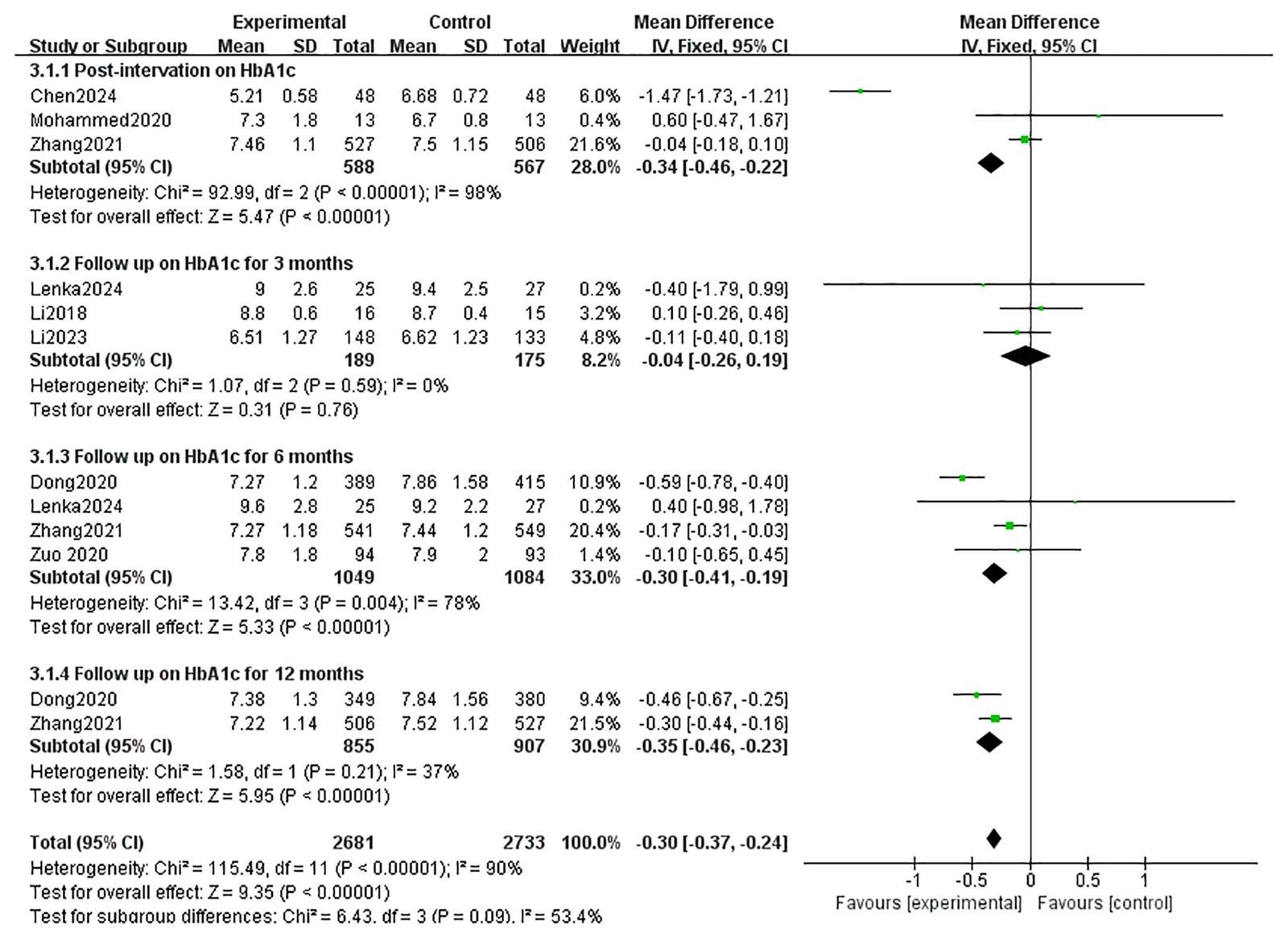
Forest plot of CBT effect on HbA1c by follow-up time. CBT = cognitive behavioral therapy, CI = confidence intervals, SD = standard deviation.

#### 3.4.3. Effect of CBT on BMI

Pooled results from 4 studies (n = 429) indicated that CBT intervention did not have a statistically significant effect on BMI levels compared to control, which may be related to the small number of included literature (SMD = −0.12; 95% CI = −0.72 to 0.48, *P* = .69, *I*^2^ = 0%; Fig. 6).^[27,31,34,35]^

**Figure 6.**
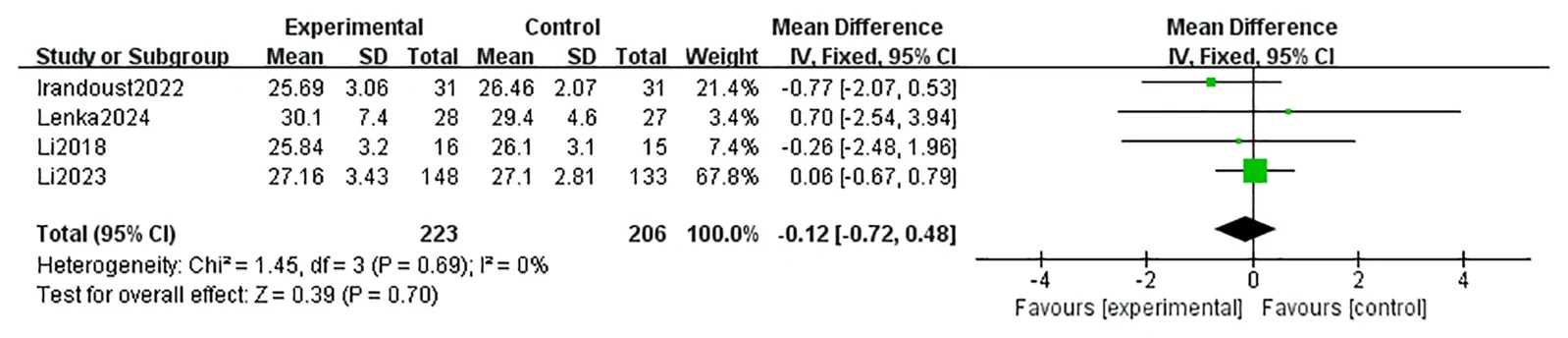
Forest plot of effect of CBT intervention on BMI. BMI= body mass index, CBT = cognitive behavioral therapy, CI = confidence intervals, SD = standard deviation.

#### 3.4.4. Effect of CBT on insomnia severity

Two studies (n = 81) assessed the effect of CBT intervention on insomnia severity using the Insomnia Severity Index (ISI).^[20,27]^ The pooled results indicated a statistically significant effect of CBT in reducing insomnia severity compared to control (SMD = −2.06; 95% CI = −4.24 to −0.12; *I*^2^ = 88%). Due to the limited number of studies (n = 2), sensitivity analysis by removing individual studies to test heterogeneity was not feasible (Fig. 7).

**Figure 7.**
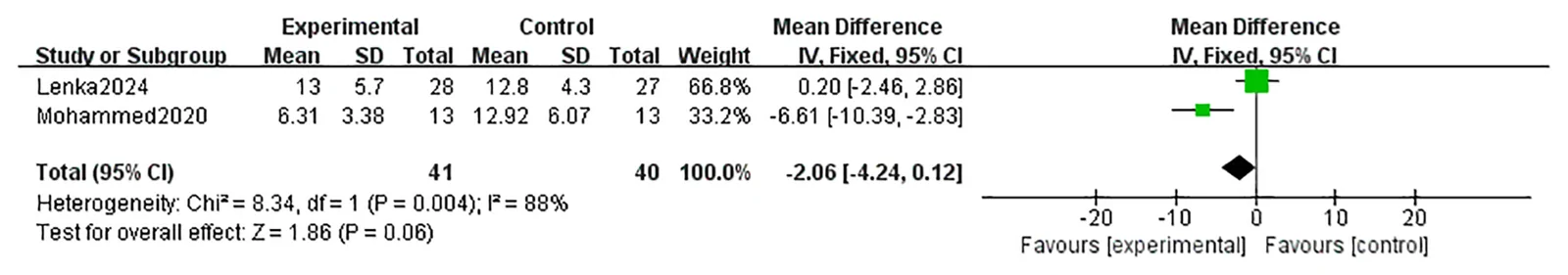
Forest plot of effect of CBT intervention on insomnia severity. CBT = cognitive behavioral therapy, CI = confidence intervals, SD = standard deviation.

#### 3.4.5. Sensitivity analysis

Leave-one-out analysis was conducted by removing each trial, demonstrating no substantial change in the effect size. Point estimates ranged from −0.53 (95% CI = −1.30 to −0.44) to −1.16 (95% CI = −1.16 to −0.84). No individual trial exerted statistically significant influence on the overall pooled result. The results are shown in Figure 8.

**Figure 8.**
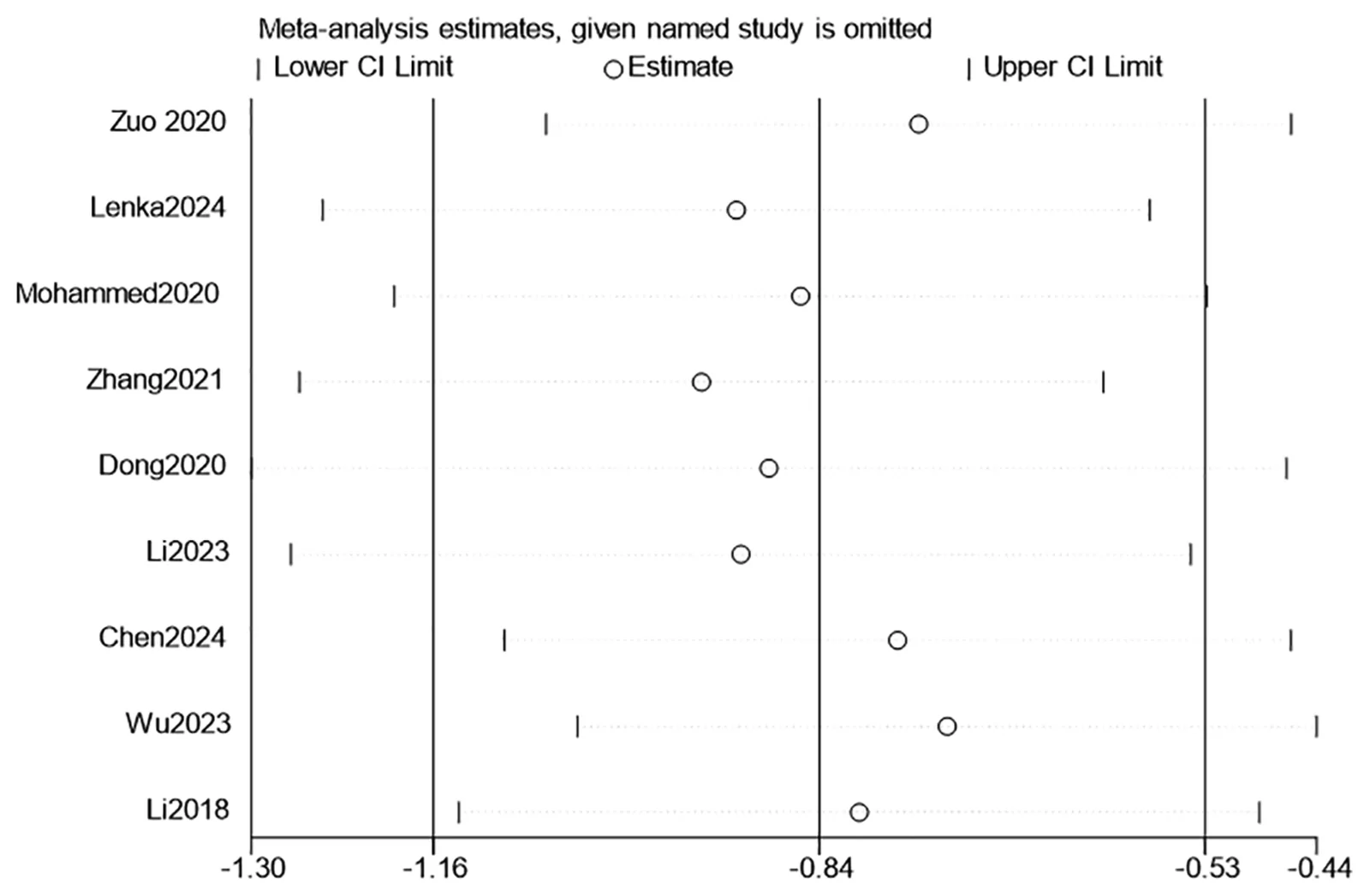
Sensitivity analysis. CI = confidence interval.

#### 3.4.6. Publication bias

Through Egger test and Begg test on sleep quality, it was found that there was no significant publication bias in Egger test (*t* = −1.97; *P* = .089) and Begg test (*P* = .9170). The results are shown in Figures 9 and 10.

**Figure 9.**
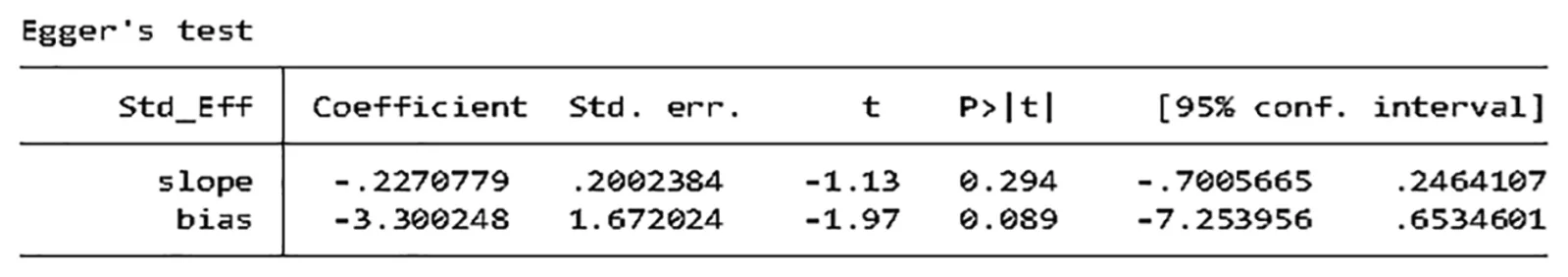
The Egger test for sleep quality.

**Figure 10.**
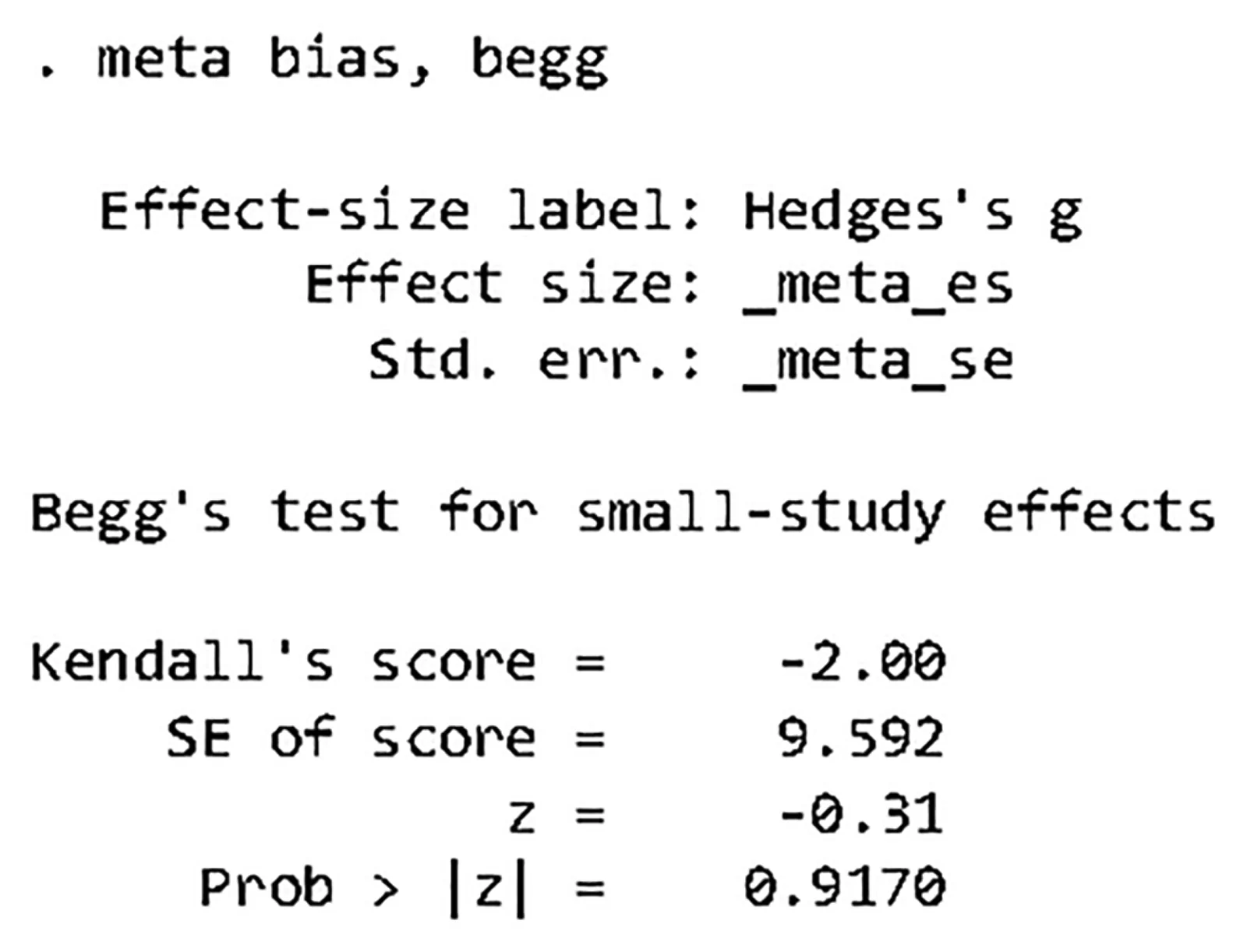
The Begg test for sleep quality.

#### 3.4.7. Meta-regression

Meta-regression results showed no statistically significant associations between the effect size (sleep quality) and disease type (*P* = .159), sample size (*P* = .829), or intervention (*P* = .627). The results are shown in Figure 11.

**Figure 11.**
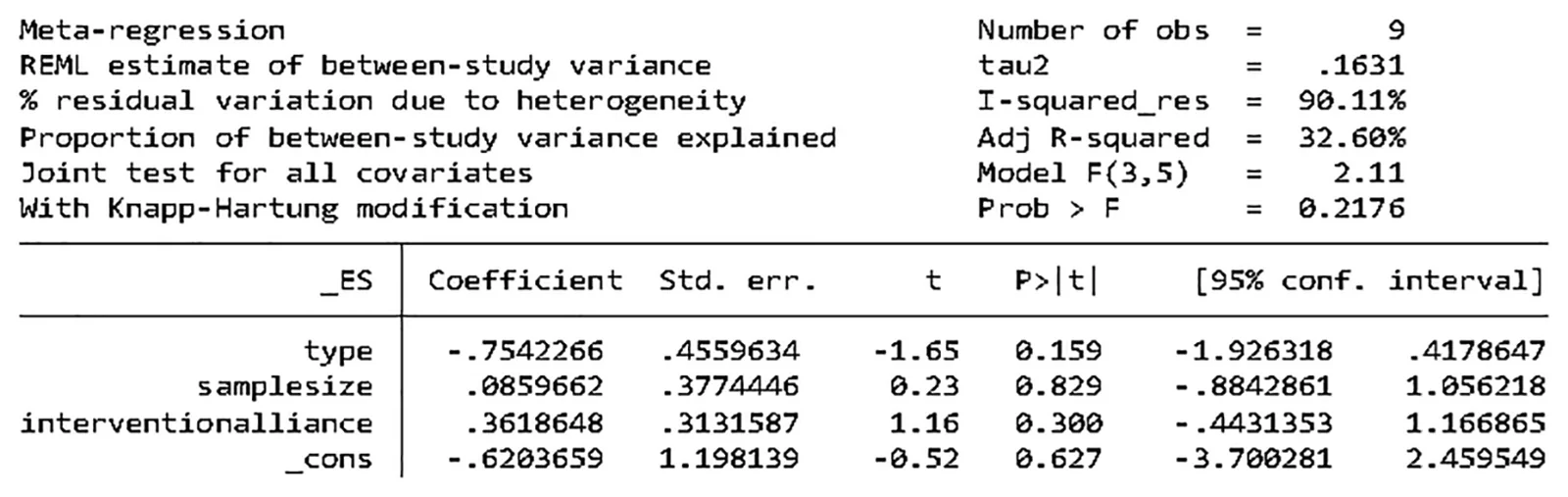
Meta regression of sleep quality.

## 4. Discussion

### 4.1. Summary of main findings

This study included 2827 participants from4 countries.^[26-35]^ The results demonstrate that CBT has a significantly positive effect on improving sleep quality and key metabolic indicators in people with diabetes. The specific manifestations are as follows: sleep quality was significantly improved. Compared to usual care or health education, CBT showed a notable effect size (mean difference [MD] = −1.28, 95% CI = −1.54 to −1.06, *P* < .001). All included studies reported statistically significant improvements, underscoring the core value of CBT for managing sleep disturbances in diabetes. In terms of blood glucose control, Significant reduction was found (MD = −0.35%, 95% CI = −0.44 to −0.25, *P* < .00001), supporting the indirect beneficial effect of CBT on metabolic function. The degree of insomnia was significantly reduced (MD = −2.06, 95% CI = −4.24 to −0.12, *P* = .004), further validating the alleviation of sleep-related symptoms. BMI showed no significant improvement (MD = −0.12, *P* = .69), potentially due to small sample size, variability in intervention duration, or the multifactorial nature of BMI (diet, exercise).

Prediction intervals are reported for all primary outcomes to contextualize the extreme statistical heterogeneity (*I*^2^ > 75% for all sleep and glycemic endpoints). All concluding statements are tempered to reflect low-to-moderate GRADE evidence certainty, avoiding overly definitive causal claims. High heterogeneity (*I*^2^ > 75% for all outcomes) and potential publication bias noted in exploratory analysis were observed. However, the core finding for sleep quality remained significant after adjustment for publication bias (MD = -1.14, *P* < .001), supporting the robustness of the conclusion.

### 4.2. Comparison and extension of existing research

Previous research has confirmed CBT’s effectiveness for sleep in diabetes but had limitations: focusing only on sleep-related outcomes, such as sleep duration and sleep diary, but not including metabolic indicators such as blood glucose and BMI, and using incomplete database searches. By including multi-dimensional outcomes (HbA1c, BMI) and searching 10 databases, this study provides stronger evidence for the comprehensive benefits of CBT, filling the gap regarding the “sleep-metabolism” link. Furthermore, the observed effect size for sleep (MD = −1.28) was larger than in previous meta-analyses, possibly due to more standardized interventions or larger sample sizes in recent studies.^[36]^

### 4.3. Analysis of potential mechanisms

The beneficial effects of CBT on both sleep and glycemia can be explained through multiple mechanisms. In terms of cognitive emotion regulation, CBT helps identify and modify maladaptive cognitions (e.g., “I must get 8 hours sleep”) and disease-related anxieties (e.g., “Any glucose fluctuation means I’m losing control”), reducing negative emotions that interfere with sleep. Reducing stress responses lowers cortisol levels, indirectly improving insulin sensitivity.^[8,37]^ Meanwhile, CBT improves poor sleep habits. Strategies like sleep hygiene education, stimulus control, and sleep restriction directly target poor sleep habits. Enhanced understanding of the diabetes-sleep link improves self-management (e.g., diet, exercise adherence)and facilitates optimized behavioral interventions.^[38]^ Regulation of physiological pathways: improved sleep can reduce chronic inflammation (e.g., IL-6, TNF-α).^[39]^ The stress response alleviated by cognitive behavioral therapy (CBT) further alleviates metabolic disorders, forming a virtuous cycle of “Improving sleep to Reduce Stress and Improve Blood Glucose Control.”

Variability in CBT intervention composition (online vs in-person, standalone vs. adjunctive therapy) is identified as a key source of clinical heterogeneity across trials, consistent with subgroup analysis results presented in Section 3.4.

### 4.4. Clinical implications of subgroup analyses

The subgroup analysis in this review demonstrated significant improvements in sleep quality observed immediately after intervention, as well as at 3, 6, and 12 months. The enhanced effects remained substantial at 6 and 12 months, suggesting long-term benefits that may be linked to the persistence of behavioral changes. Both short-term (≤6 weeks) and extended (>6 weeks) intervention protocols showed marked improvements in sleep quality, with no statistically significant differences between them. This design offers clinical flexibility: short-term programs deliver quick results, while long-term approaches better sustain effects and allow customization based on patient needs. Subgroup analysis revealed that disease subtypes (e.g., isolated diabetes, diabetes with insomnia/comorbidities) significantly influenced outcomes, indicating that CBT protocols may need optimization for specific subpopulations (e.g., enhanced psychological support for those with complications)^.[40]^

The 0.30% to 0.35% absolute HbA1c reduction observed in pooled analyses is clinically meaningful relative to baseline glycated hemoglobin levels documented in included trials and comparable to small-to-moderate effect magnitudes from standard diabetes lifestyle modification interventions. Treatment benefits persist through 12-month follow-up, demonstrating durable metabolic improvements alongside sleep gains.

### 4.5. Limitations of the study

This review synthesizes the impact of cognitive behavioral therapy on sleep quality and blood glucose control in all diabetes subtype patients. The included RCTs provide robust evidence for the clinical application of cognitive behavioral therapy in sleep management. Additionally, it enhances healthcare professionals’ work capabilities. However, this systematic review and meta-analysis has certain limitations. First, meta-regression failed to link heterogeneity to variables like gender ratio or intervention frequency. Unmeasured factors (e.g., therapist qualifications, cultural differences, variable CBT co-interventions) likely contribute to the extreme inter-study heterogeneity observed. Second, 80% of studies had unclear/high risk concerning allocation concealment. Only 2 studies blinded outcome assessors. Blindness of subjects was not possible due to the intervention protocol. Performance bias is inherent in CBT trials. Third, uneven distribution of outcome studies. Analyses for BMI (n = 4) and insomnia severity (n = 2) had limited statistical power to detect small treatment effects. Large sample size disparities (28–1142) amplified heterogeneity across pooled estimates. Finally, only articles published in English or Chinese were reviewed, which may lead to potential selection bias, and therefore the generalizability of the results may be limited.

Conclusions are framed cautiously throughout, acknowledging the high I^2^ values, limited number of trials for secondary outcomes, and moderate/low GRADE evidence certainty as core limitations.

### 4.6. Future research directions

In future large-scale multicenter RCTs, standardized cognitive behavioral therapy protocols (including uniform core modules and fixed treatment courses) should be implemented with strict adherence to allocation concealment and blinding of outcome assessors. Stratified analyses should be conducted based on intervention details (module combinations, dosage, delivery format) and patient characteristics (age, diabetes subtype, severity of comorbidities) to identify modulating factors affecting CBT efficacy. The follow-up period should be extended with expanded evaluation metrics, including long-term follow-up (≥2 years) to assess treatment sustainability. Additional assessment dimensions should incorporate metabolic indicators (fasting blood glucose, lipid levels) and psychological measures (depression scales). Particular emphasis should be placed on evaluating CBT’s effectiveness in high-risk populations such as gestational diabetes patients, patients with severe diabetic complications, and elderly adults living with diabetes.

## 5. Conclusion

This systematic review and meta-analysis provides evidence that Cognitive Behavioral Therapy significantly improves sleep quality, reduces HbA1c levels, and alleviates insomnia severity in people with diabetes. Both short-term and longer-term interventions demonstrate measurable benefits. Despite prominent limitations including extreme inter-study statistical heterogeneity, variable methodological quality across trials, and moderate-to-low GRADE evidence certainty, CBT holds significant clinical value as a non-pharmacological intervention within comprehensive diabetes management. Future high-quality standardized RCTs are needed to optimize CBT treatment protocols, define targeted diabetes subpopulations, and facilitate wider clinical implementation of sleep-focused behavioral interventions.

## Acknowledgments

The authors gratefully acknowledge the financial supports.

## Author contributions

**Conceptualization:** Yuxiao Jiao, Lanyu Zhu.

**Data curation:** Yuxiao Jiao.

**Formal analysis:** Yuxiao Jiao, Yueqi Yin.

**Funding acquisition:** Lanyu Zhu.

**Investigation:** Yuxiao Jiao, Xiaofei He, Lulu Ma, Bo Zhang.

**Methodology:** Yuxiao Jiao, Xiaofei He.

**Resources:** Yuxiao Jiao.

**Software:** Yuxiao Jiao, Xiaofei He, Lulu Ma.

**Writing – original draft:** Yuxiao Jiao.

**Writing – review & editing:** Yuxiao Jiao, Lanyu Zhu.
